# Permanent Peripheral Nerve Stimulation for Chronic Cervical Neck Pain: A Case Report

**DOI:** 10.7759/cureus.99344

**Published:** 2025-12-15

**Authors:** Philopateer Messeha, Grant Weiderman, Jake Belli, Shivang Patel, Tony El-Hayek

**Affiliations:** 1 Osteopathic Medicine, Lake Erie College of Osteopathic Medicine, Bradenton, USA; 2 Pain Medicine, Mercy Health Allen Hospital, Oberlin, USA

**Keywords:** cervical neck pain, epidural injection, neuromodulation, peripheral nerve stimulation, radiofrequency ablation

## Abstract

Chronic cervical neck pain is a prevalent and debilitating condition that can be difficult to treat, particularly when patients fail to respond to conservative approaches, including physical therapy, medications, and epidural injections. We present the case of a 46-year-old female with a 2-year history of chronic left-sided neck pain and intermittent radicular symptoms, refractory to multiple pharmacologic and interventional treatment options. Imaging demonstrated degenerative disc disease and severe foraminal narrowing. The patient underwent a trial of left-sided cervical peripheral nerve stimulation, resulting in a 50% reduction in pain initially and a 90% reduction at the time of lead removal. This led to a plan for permanent implantation. This case highlights the potential of peripheral nerve stimulation as an effective intervention for chronic cervical pain, especially in patients who are not candidates for surgery or have failed other treatment modalities.

## Introduction

Cervical neck pain is a prevalent and often debilitating condition that negatively impacts quality of life and functional ability. Epidemiological data show that neck pain affects over 30% of individuals at some point in their lives and has been attributed to the fourth leading cause of disability worldwide [1]. This burden is significant in the aging population and those engaged in sedentary or repetitive work, with varying prevalence rates based on demographic, psychosocial, and occupational factors [2]. The etiology of cervical neck pain is multifactorial, involving musculoskeletal and neurologic components. Common causes include cervical muscle weakness, spondylosis, stenosis, or herniation. Cervical pain can present in two forms: axial or radicular. Axial neck pain presents with pain that is localized in the cervical spine region and can spread to the shoulders or upper back. Radicular neck pain radiates into the upper limb and can be accompanied by numbness or weakness due to nerve compression [3]. While pharmacological approaches remain a primary intervention in the treatment of cervical neck pain, radicular symptoms refractory to conservative management are often treated with surgical intervention, which has demonstrated improvements in pain and function for many patients, although long-term outcomes can vary [4]. 

Epidural steroid injections are another interventional treatment option to provide short-term pain relief, but should be avoided in patients with worsening motor weakness, high-grade spondylolisthesis, and spinal stenosis [5]. Radiofrequency ablation (RFA) is another viable non-surgical option for managing cervical facet-mediated pain. RFA is a process that generates heat through high-frequency electrical currents and damages nerve fibers to interrupt pain signal transmission. Relief can last up to 12-24 months in a responsive patient [6]. According to the clinical guidelines set by the American Society of Regional Anesthesia and Pain Medicine and the American Academy of Pain Medicine, a positive response to two diagnostic median branch nerve blocks is required before using RFA [7]. For patients who have not achieved relief with conservative measures or more aggressive therapies like RFA, peripheral nerve stimulation (PNS) has become an increasingly popular approach. PNS delivers a controlled electrical impulse to a peripheral nerve and activates fast-conducting nerve fibers to reduce chronic pain. Repeated stimulation causes an alteration in how the brain perceives pain, which ultimately decreases pain perception [8]. Here, we present the case of a 46-year-old female refractory to conservative approaches who developed significant symptomatic relief after implementation of PNS.

## Case presentation

**Figure 1:**
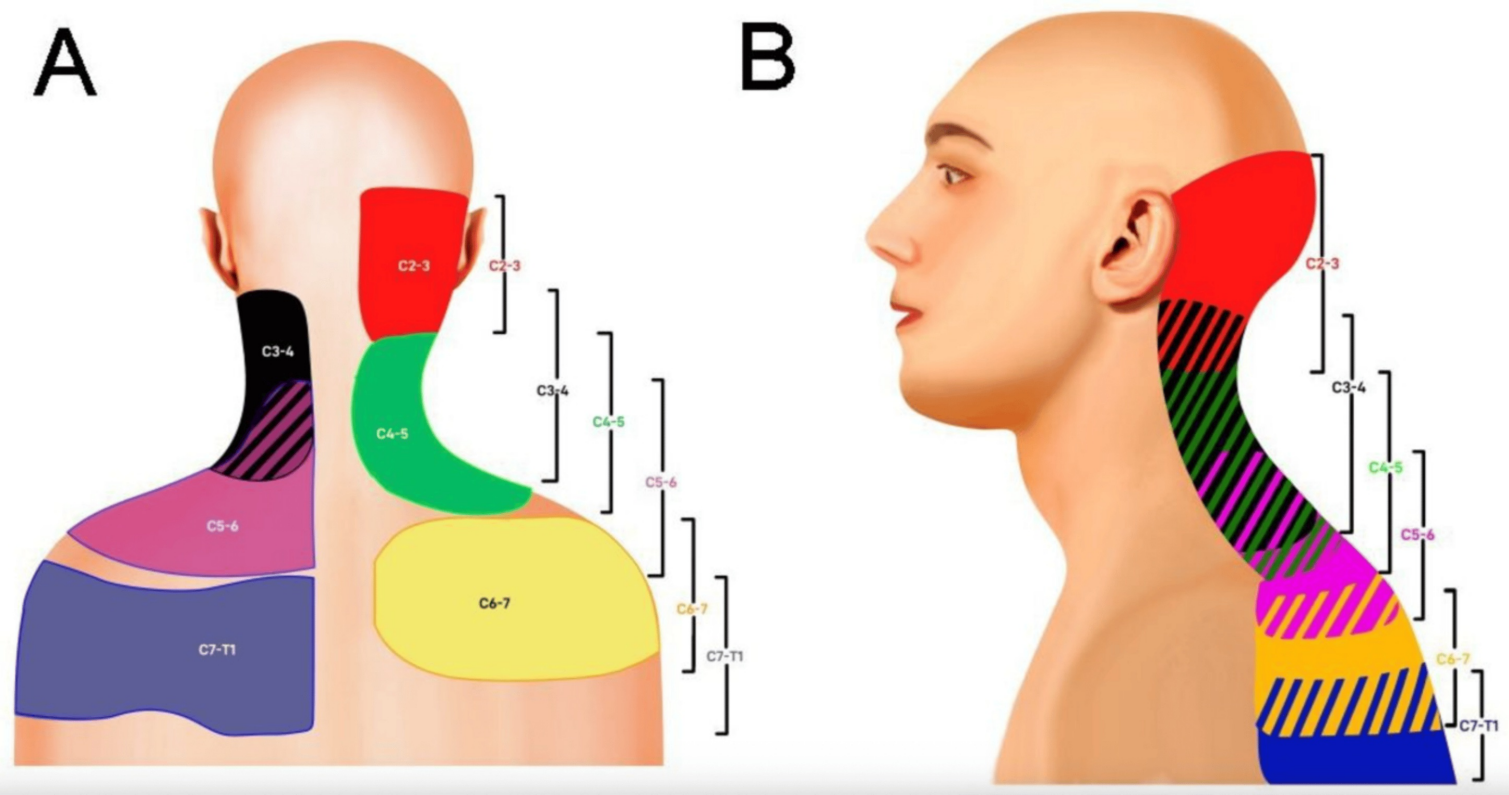
Posterior (A) and lateral (B) segmental maps illustrating referral patterns from the cervical facet joints (C2–3, red; C3–4, black; C4–5, green; C5–6, purple; C6–7, yellow; C7–T1, blue). Striped regions indicate areas of overlap between adjacent facet joint referral zones. Source: Hurley et al. [7]. Licensed under Creative Commons Attribution 4.0 Unported (CC BY 4.0) - https://creativecommons.org/licenses/by/4.0/.

A 46-year-old female with a 2-year history of chronic left-sided neck pain with occasional radiation into the left trapezius presented to the pain clinic with worsening symptoms. Her pain distribution, characterized by left-sided neck pain with trapezial radiation, overlapped most closely with the referral zone of the C5-6 facet joint, as illustrated on the cervical facetogenic pain referral map shown in Figure 1. The pain exacerbations were accompanied by headaches, neck stiffness, and numbness. Symptoms were exacerbated by neck extension and rotation. The patient reported a significant decline in her ability to carry out activities of daily living (ADLs) and rated her pain a 7 out of 10 according to the visual analog scale (VAS) score, significantly limiting her mobility and negatively affecting her quality of sleep.

Her previous medication interventions included neuropathic medications and muscle relaxants such as gabapentin, pregabalin, duloxetine, and tizanidine. Procedures such as cervical RFA on the left side at C5-C6 and C6-C7 levels and multiple epidural steroid injections all failed to provide pain relief. 

On a physical exam, the patient exhibited focal tenderness over the left cervical paraspinal musculature, numbness, and a positive Spurling’s test on the left. Cervical range of motion was limited, particularly with extension and leftward rotation, which reproduced her pain. Palpation revealed increased muscle tension in the left trapezius. Motor strength was 5/5 in all major muscle groups, and sensory function was intact throughout.

**Figure 2:**
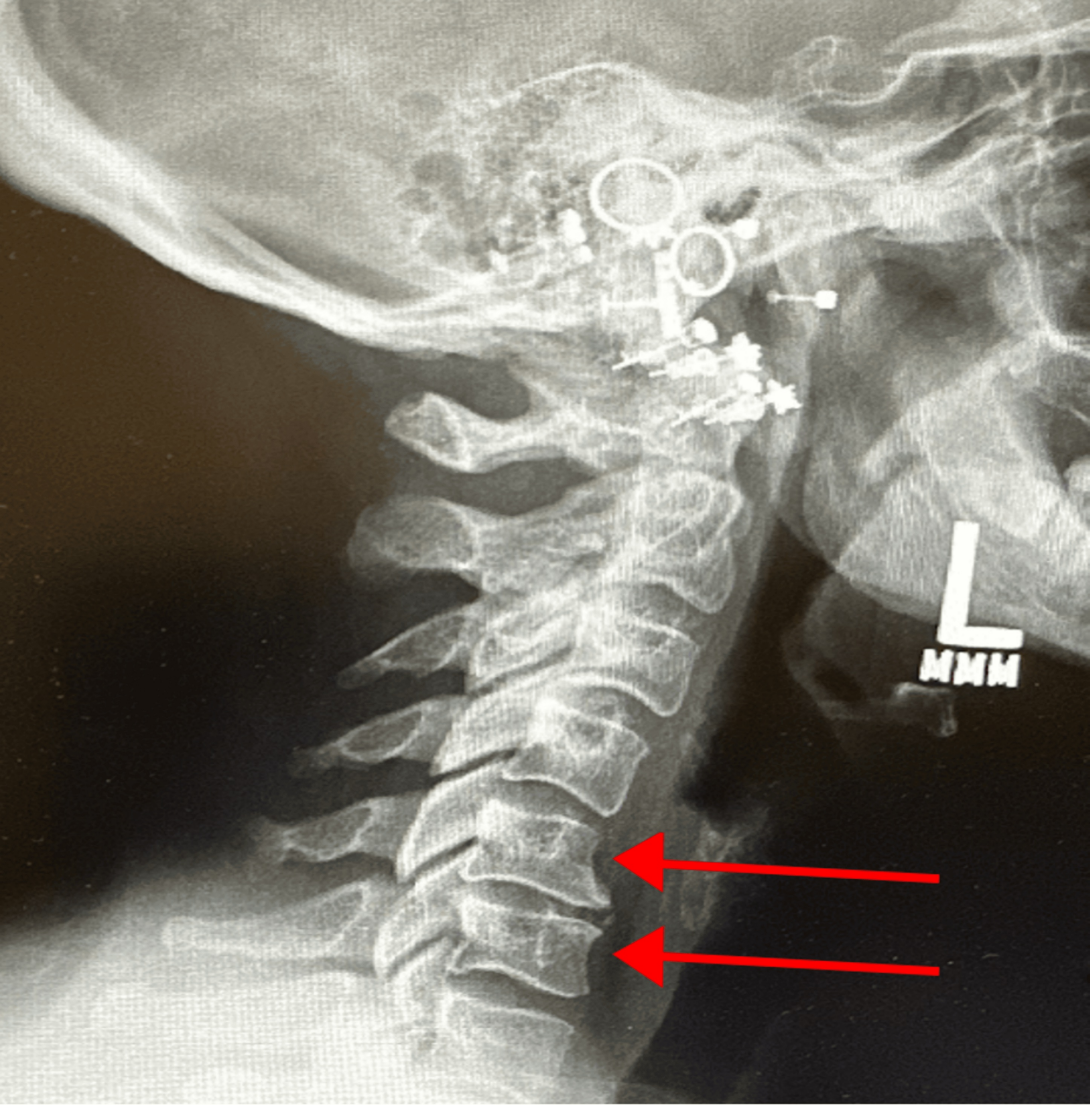
Lateral cervical radiograph highlighting C5–C6 degenerative disc disease and reversal of lordosis (red arrows) L: Left

**Figure 3:**
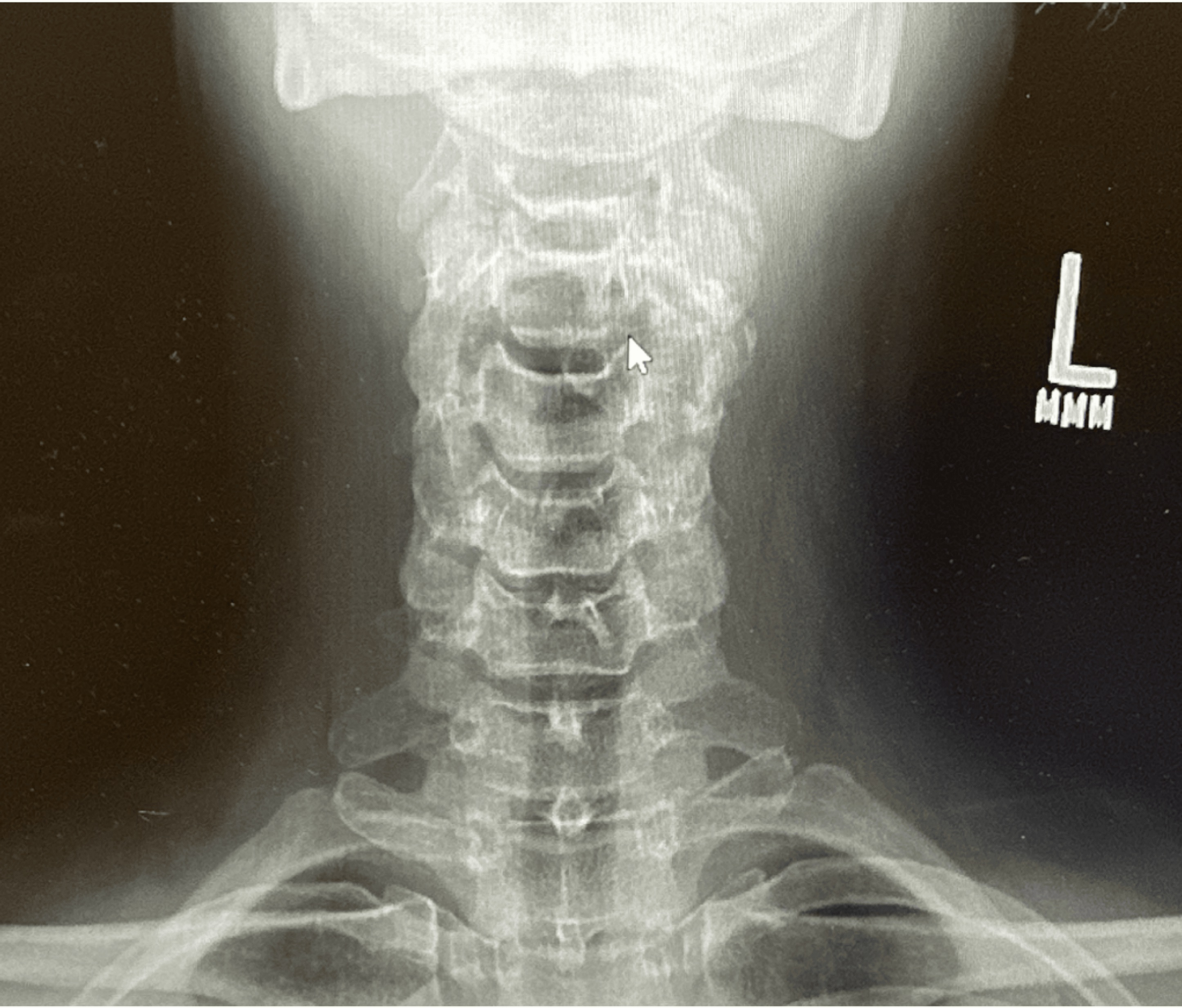
Posterior cervical X-Ray showing degenerative changes at C5–C6 with reversal of cervical lordosis L: Left

**Figure 4:**
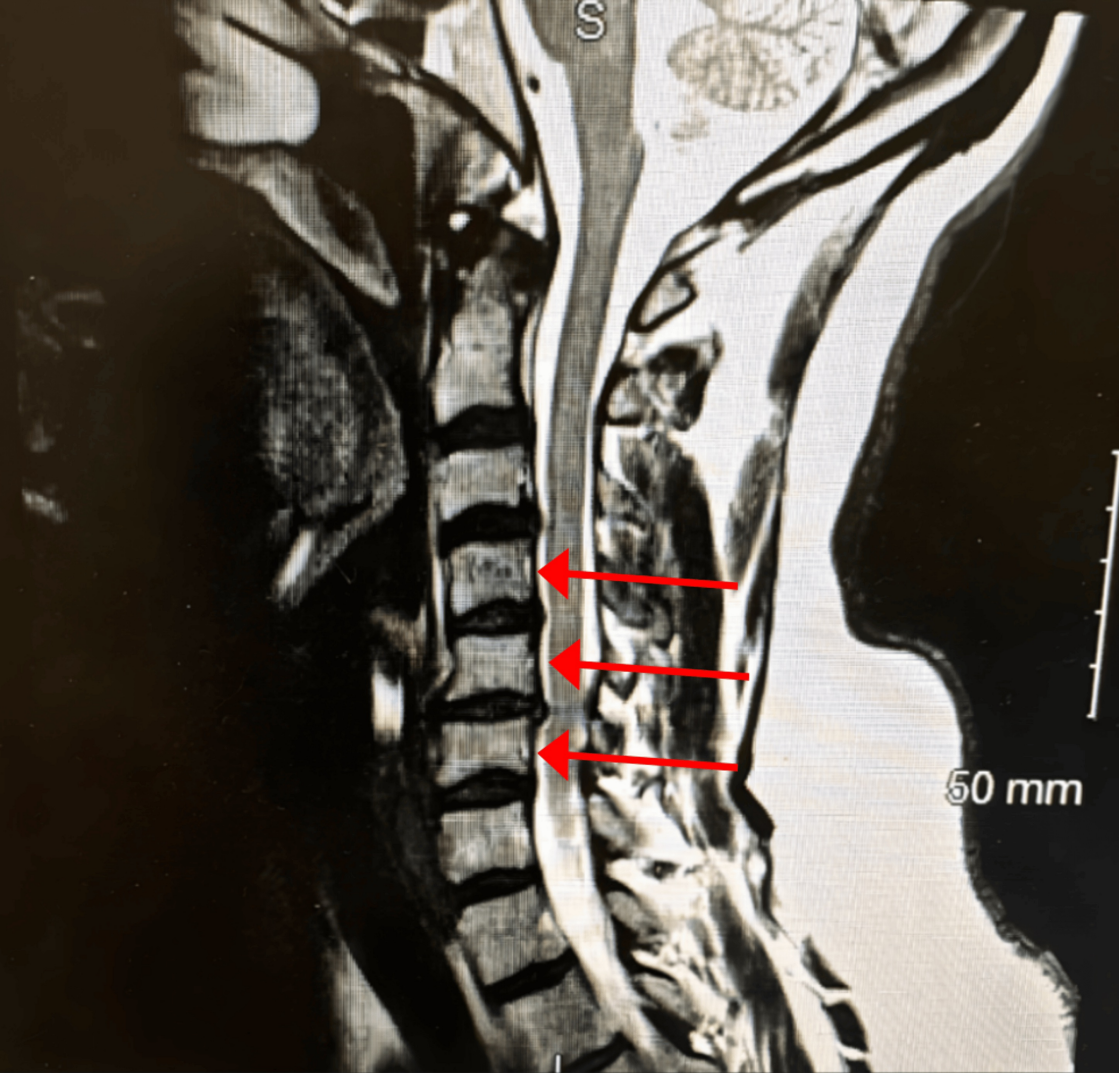
Sagittal MRI of the cervical spine demonstrating multilevel degenerative changes (red arrows) S: Superior

Cervical X-rays demonstrated degenerative disc disease at C5-C6 and a reversal of cervical lordosis, likely secondary to muscle spasm, as shown in Figure 2 and Figure 3. MRI of the cervical spine revealed severe left foraminal narrowing at C3-C4, mild central canal stenosis at C4-C5, and severe bilateral foraminal narrowing at C5-C6, which was more pronounced on the left as shown in Figure 4.

Given the severity of her symptoms and lack of response to prior interventions, the patient underwent left cervical PNS on April 3rd, 2025. The PNS was performed under ultrasound guidance. After identifying the left C5-C6 cervical medial branch trajectory, a percutaneous needle was advanced to the target nerve. Proper placement was confirmed by reproducing paresthesia over the patient’s painful region. She reported a 50% reduction in pain following the procedure. The temporary PNS lead was removed on May 29th, 2025, with a 90% reduction in pain. Given the significant improvement in symptoms, plans were made for permanent cervical PNS implantation on June 2nd, 2025. 

Following placement of the permanent cervical PNS implant, she reported approximately 90% pain relief in her neck at her two-week follow-up, with marked improvement in mobility and daily activities. She was able to return to her baseline functional level. At her two-month follow-up, the patient continued to experience similar pain relief and functional gains, with decreased reliance on analgesic medications.

## Discussion

Chronic cervical neck pain is considered a mechanical pain syndrome due to changes in the structure of the spine. The pathophysiology is often the breakdown of ligamentous structures from osteoarthritis or another inflammatory condition that leads to the sensitization of nociceptive pain fibers. The pain is localized to the specific area of damage and does not radiate or cause any neurologic deficits. In contrast, cervical radiculopathy is characterized by neuropathic pain and sensorimotor deficits due to compression or inflammation of the cervical nerve root. This often occurs due to disc herniation, osteophyte formation, or spondylosis. This will lead to pain radiating in a dermatomal distribution and have accompanying neurologic deficits such as paresthesia, weakness, and numbness in the corresponding myotome [9]. This patient met the criteria for chronic cervical neck pain without evidence of radiculopathy, as evidenced by her CT and MRI. Conservative management is first-line treatment for cases like this, while surgery is reserved for patients with radiculopathy or neurological compromise [10]. Despite multiple conservative therapies, including neuropathic medications, epidural injections, and RFA, our patient continued to experience significant pain and functional limitations, indicating the need for alternative treatment options like PNS. 

PNS provides analgesia through complementary peripheral and central mechanisms that modulate pain signaling at multiple levels of the nervous system. At the site of stimulation, PNS activates large-diameter, low-threshold A-β fibers, which normally transmit non-painful mechanosensory input. Recruitment of these fibers excites inhibitory interneurons within the dorsal horn of the spinal cord. These inhibitory interneurons suppress nociceptive transmission from smaller A-δ and C fibers, thereby reducing the relay of painful stimuli to higher centers. This gate control mechanism decreases ascending nociceptive signaling before it can reach the brain’s pain-processing regions [8,11]. Beyond this segmental inhibition, PNS exerts broader central effects. Stimulation can reduce dorsal horn hyperexcitability and modulate ascending sensory pathways, including the medial lemniscal system, which relays somatosensory information to the thalamus and cortex. PNS also engages descending inhibitory pathways originating from supraspinal centers, including the periaqueductal gray and rostroventral medulla. These descending systems utilize serotonergic (5-HT2, 5-HT3), GABAergic, and glycinergic neurotransmission to suppress dorsal horn activity further, leading to a durable reduction in central pain sensitization [12]. Unlike RFA or invasive surgery, PNS is a nondestructive therapy that could potentially alleviate chronic pain. A recent case series targeting the cervical medial branch nerve demonstrated a significant reduction in pain intensity and disability after 60 days of implantation, supporting the role of PNS as a minimally invasive, reversible treatment option in patients [13]. In a different 12-month COMFORT-randomized controlled trial, PNS showed an 87% response rate and an average pain reduction of 69% at one year among patients with chronic neuropathic pain. Although the device was used on the lower back, shoulder, knee, and foot/ankle, these findings suggest promising potential for patients with neck pain [14].

PNS offers a minimally invasive, reversible option that can be particularly beneficial for patients who are poor surgical candidates or who have failed to respond to conservative treatment methods adequately. In this patient, PNS was an appropriate treatment choice given her refractory pain and lack of surgical candidacy. The targeted neuromodulation achieved meaningful and sustained pain relief, significantly improving her quality of life and functional capacity without the complications associated with operative management. These outcomes support the expanding role of PNS in treating chronic cervical pain and emphasize the need for more controlled studies for more refined patient selection criteria and optimized stimulation parameters.

## Conclusions

Chronic cervical neck pain is a common complaint of many patients and poses a significant challenge to treat, especially when standard conservative measures and interventional procedures fail to provide pain relief. This case highlights the effectiveness of peripheral nerve stimulation in a patient with severe, treatment-resistant cervical pain. Following a successful trial period, the patient had marked improvement in symptoms, which supports the use of PNS as a viable and minimally invasive treatment option as an alternative to surgery. While further research is needed to establish the long-term efficacy of PNS, this report contributes to the existing evidence that PNS can be utilized to decrease chronic pain refractory to conventional therapies.
